# Group Decisions in Biodiversity Conservation: Implications from Game Theory

**DOI:** 10.1371/journal.pone.0010688

**Published:** 2010-05-27

**Authors:** David M. Frank, Sahotra Sarkar

**Affiliations:** 1 Department of Philosophy, University of Texas, Austin, Texas, United States of America; 2 Section of Integrative Biology and Department of Philosophy, University of Texas, Austin, Texas, United States of America; University of Bern, Switzerland

## Abstract

**Background:**

Decision analysis and game theory [1], [2] have proved useful tools in various biodiversity conservation planning and modeling contexts [3]–[5]. This paper shows how game theory may be used to inform group decisions in biodiversity conservation scenarios by modeling conflicts between stakeholders to identify Pareto–inefficient Nash equilibria. These are cases in which each agent pursuing individual self–interest leads to a worse outcome for all, relative to other feasible outcomes. Three case studies from biodiversity conservation contexts showing this feature are modeled to demonstrate how game–theoretical representation can inform group decision-making.

**Methodology and Principal Findings:**

The mathematical theory of games is used to model three biodiversity conservation scenarios with Pareto–inefficient Nash equilibria: (i) a two–agent case involving wild dogs in South Africa; (ii) a three–agent raptor and grouse conservation scenario from the United Kingdom; and (iii) an *n*–agent fish and coral conservation scenario from the Philippines. In each case there is reason to believe that traditional mechanism–design solutions that appeal to material incentives may be inadequate, and the game–theoretical analysis recommends a resumption of further deliberation between agents and the initiation of trust—and confidence—building measures.

**Conclusions and Significance:**

Game theory can and should be used as a normative tool in biodiversity conservation contexts: identifying scenarios with Pareto–inefficient Nash equilibria enables constructive action in order to achieve (closer to) optimal conservation outcomes, whether by policy solutions based on mechanism design or otherwise. However, there is mounting evidence [6] that formal mechanism–design solutions may backfire in certain cases. Such scenarios demand a return to group deliberation and the creation of reciprocal relationships of trust.

## Introduction

Efforts to conserve and promote biodiversity require at least two normative commitments [7]. *First*, operationalizing the concept of “biodiversity” involves deciding which taxa or other biodiversity surrogates are worth the allocation of finite conservation resources [7]–[9]. *Second*, the goal of biodiversity conservation must be negotiated with other normatively salient social goals such as economic well–being, public health, *etc*., especially when land use policies are being formulated [7], [10], [11]. In both cases, there is ample potential for conflict. When these conflicts occur for a single agent (individual or organized group), decision support tools based on multi–criteria analysis (MCA) typically provide useful insight [3], [12], [13]. In this paper we show that when these conflicts involve differences between more than one agent, game theory may play a similar role. The mathematical theory of games has traditionally been used by social scientists to model strategic interaction, and is thus easily adapted to modeling conflicts between multiple stakeholders in conservation contexts. The main result in this paper is to use game theory to show that there exist conservation conflicts with Pareto–inefficient Nash equilibria. The games we analyze share this property with the well-known “Prisoner's Dilemma” (PD) and many other games [14].

In biodiversity conservation contexts, two potential roles for game theory can be distinguished. The first role, well–understood in evolutionary theory and economics, is *descriptive*. Evolutionary games can be used to model frequency–dependent selection [15], [16]. In economics, traditional (“rational choice”) game theory can be used to explain macro–behavioral outcomes by appealing to the equilibrium of some underlying game [1], [2]. Game theory can be used in the same way to describe biodiversity conservation conflicts [5]. However, our focus will be on the second *normative* (or prescriptive) role of game theory. We show that identifying conflicts with Pareto–inefficient Nash equilibria enables constructive action in order to achieve (closer to) optimal conservation outcomes, whether by familiar policy solutions based on mechanism design or otherwise [6]. (A *mechanism*–*design* solution is one which is based on the design of optimal individual incentive structures.) Attaining Pareto–efficient cooperative outcomes need not proceed via formal institutional arrangements at all, but may be achieved through deliberation and the creation of reciprocal relationships of trust and other confidence–building measures. Moreover, there is reason to suggest that, in certain cases, mechanism–design solutions may backfire. Game theory thus serves as a normative tool, and provides a precise analytical framework which can be used to recognize the sub–optimality of certain conservation situations relative to a well–defined set of assumptions, while pointing towards possible solutions.

The Methods section introduces decision analysis, game theory, and describes one well–studied game with a Pareto–inefficient Nash equilibrium (the Prisoner's Dilemma). In the Results and Discussion section we report two–agent, three–agent, and *n*–agent conservation conflicts from case studies. Throughout this paper, we will use the following standard definitions of Nash equilibrium and Pareto–efficiency. An outcome is a *Nash equilibrium* if no agent can do better by *unilaterally deviating* from the current course of action (strategy): each agent's action is a “best response” to the actions of the other agents. An outcome is *Pareto*–*efficient* if, relative to the other possible outcomes, no agent can be made better off without making at least one agent worse off. An outcome is *Pareto*–*inefficient* if there exists some other outcome such that at least one agent is made better off while no agent is made worse off.

## Results

### Two–agent conservation conflict: Wild dogs in South Africa

In South Africa, endangered carnivorous wild dogs (*Lycaon pictus*) were re-introduced into conservation areas in 1980–1981, and again in 1997 and in the early 2000s [17], [18]. The conservation plan analyzed here involved re-introduction of the species to the 900 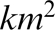 Hluhluwe-iMfolozi Park in eastern South Africa [19], notable for attracting many South African and international visitors, primarily ecotourists. The park contained numerous large carnivores, including spotted hyaena (*Crocuta crocuta*), black-backed jackal (*Canis mesomelas*), cheetah (*Acinonyx jubatus*), lion (*Panthera leo*), and leopard (*Panthera pardus*). Conservation proponents intended to create meta–populations of *Lycaon pictus* that would be managed with occasional translocation between sub–populations to facilitate gene flow [20]. By 2004, after more than 20 years of sporadic conservation measures, it was reported that the park itself supported nearly 50 dogs living in six packs, with an unknown number living in the surrounding unprotected areas.

Both biodiversity conservation proponents, concerned that only about 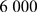 individuals of this species remained in the wild, and the ecotourism industry, which found that tourists rated seeing the wild dogs quite highly, had an interest in promoting the re-introduction and translocation policies [21]. However, rural herders and game farmers had an interest in the safety of their livestock or game populations, and many of them adopted a policy of killing wild dogs and other carnivores that escaped from conservation areas.

Although the local farmers, herders, and gamekeepers on private land, as well as Zulu villagers on communal land, were partly protected by the Hluhluwe-iMfolozi Park's electric perimeter fence, many of the large carnivores, especially the wild dogs, were known to escape from the park. Local community members held wild dogs responsible for roughly 15% of the annual livestock loss [18]. In response, conservation proponents accompanied the re-introduction and translocation policies with a public–relations campaign and a conservation education program for surrounding communities from 1999 to 2000. Results were assessed for progam effectiveness in 2003. While ecotourists consistently reported positive attitudes toward seeing the wild dogs, and were willing to pay up to $ 150 for a chance to see them, villagers' attitudes toward the conservation program became *more* negative between 1999 and 2003. Furthermore, among those with limited educational background, misconceptions about the wild dogs and the goals of biodiversity conservation were found to be widespread, and escaped dogs continued to be occasionally killed despite legal protection.

#### Game-theoretic Analysis

The game represented in Table 1, which has the structure of a Prisoner's Dilemma (PD), can be used to represent the conflict between the conservation proponents (row) and local herders (column). We treat bodiversity conservation proponents and the ecotourism industry as one agent, 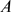, because of their common shared interest; in the analysis below they will be referred to as conservation proponents. Each action available to 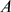 corresponds to a row of Table 1: these are to continue the re-location and translocation policy (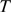) or not do so (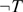). Similarly, we treat the herders and game farmers, 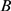, as one agent and simply refer to them jointly as local herders. The actions available to 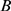 correspond to the columns: these are to have a policy of killing escaped dogs (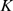) or not do so (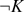). The numbers represent ordinal rankings of the outcomes, where 1 is the best outcome, 2 is the next best outcome, and so on, and are given 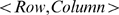 with the first entry indicating the rank for 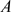 and the second the rank for 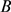. The standard assumptions of one–stage games are applicable: each agent has full knowledge of its preference structure and is a competent maximizer over its own preference ordering.

**Table 1:** Two–Agent Game with Pareto–inefficient Nash Equilibrium.

	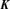	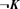
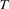	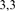	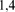
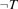	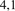	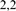

Agents: Column: local herders; Row: conservationists and eco-tourism industry. Strategies for local herders: 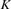: Kill escaped wild dogs (or not, 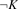). Strategies for conservationists and eco-tourism industry: 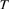: Continue re-location and translocation policy (or not, 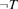). Numbers represent purely ordinal preferences over outcomes (where 1 is most preferred, 2 the next most preferred, and so on), and are given 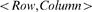.

Obviously, the best outcome for 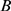 is 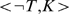, while the best outcome for 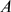 is 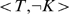. The worst outcome for 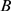 is clearly 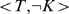, assuming the wild dogs are responsible for significant livestock loss. The worst outcome for 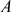 is 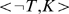, since no conservation translocation is pursued while 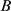
's policy threatens the feasibility of future conservation programs. The second– and third–best outcomes for 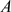 are 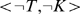 and 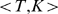, respectively, on the assumption that the translocation policy comes at significant cost, and if killing takes place the cost of the translocation program would not be worth the little conservation value it would generate. The second– and third–best outcomes for 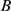 are 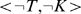 and 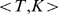, respectively, on the assumption that without a translocation policy fewer wild carnivores threaten their livestock, while killing the escaped wild dogs is itself costly.

For 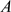, 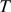 is preferred to 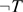, since whatever 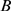
's policy, the outcomes in which translocation policies are pursued are ranked higher: 3 as opposed to 4 and 1 as opposed to 2. The same reasoning on preferences shows, for 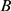, 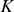 is preferred to 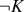. The unique pure–strategy Nash equilibrium is thus 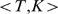, since neither agent can do better by unilaterally deviating from the strategy already being followed. (In *pure* strategies each of the agents only uses one of the available options and does not mix them in some proportion. For simplicity we do not analyze such *mixed* strategies—they are unlikely to be followed on the ground and, in this case, would not make any difference in the formal analysis.) This equilibrium outcome, however, is Pareto–inefficient, since 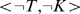 is ranked 2 for *both* agents as opposed to 3. While 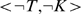 is not the unique Pareto–efficient solution, since 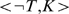 and 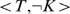 are *most* preferred by 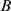 and 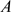, respectively, and *ipso facto* Pareto–efficient, these latter two outcomes are unattractive solutions as they are the *least* preferred by some agent.

#### Discussion: Wild dogs in South Africa

Gusset *et. al.* [18] have analyzed this conflict in some detail but did not note its relation to the Prisoner's Dilemma. Besides documenting the existence of the conflict between conservation proponents and local herders, they providede insight into possible solutions that prioritize conservation (and thus assume that 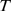 is necessarily preferred to 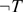). These solutions include continuing programs of conservation education, compensation measures for livestock loss, and participatory management policies [22]. Modeling the situation as a game provides additional insight. Any conservation–prioritizing solution to the conflict must either alter the payoffs for the local stakeholders (the herders), by de–incentivizing 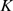 or incentivizing 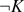, via conventional mechanism–design solutions involving (effective) law enforcement and/or financial incentives, or else directly alter the preferences of the locals, which was presumably the goal of conservation education. Gusset *et. al.* [18] noted that most of the locals had generally negative views of wild dogs. This suggests that improved husbandry practices combined with conservation education may be the most cost–effective solution.

However, Gibson and Marks [5] used a two–agent game to analyze the interactions between law enforcement personnel and wildlife hunters in Zambia. Though they did not explicitly analyze Nash equilibria or Pareto efficiency, their models revealed ample potential for conflict which was already clear on the ground. Zambia's Administrative Management Design for Game Management Areas (ADMADE) program attempted a reconciliation through the conventional mechanism–design solutions mentioned in the last paragraph except for the emphasis on conservation education in South Africa. ADMADE's attempt met with little success [5] indicating that, even in straightforward two–agent situations, mechanism–design solutions may not be effective. Prospects for such solutions are even more dim in more complex situations, to which we now turn.

### Three–agent conservation conflict: Raptors and red grouse

In Britain, in the 1990s, the relationship between raptors and their avian prey emerged as one of the more contentious issues in discussions of natural habitat conservation and management [23]–[25]. Whereas many raptor species' populations had begun to recover from their earlier pesticide–induced low levels of the 1970s, their prey species' populations were often in decline. Thirgood *et al.* [25] reviewed how this conflict was being played out in the case of the Hen Harrier (*Circus cyaneus*) and Red Grouse (*Lagopus lagopus scotius*) on heather moorlands dominated by Ling Heather (*Calluna vulgaris*). The distribution of these heather moorlands was largely limited to Britain and Ireland with smaller areas elsewhere in Europe. Consequently, in Britain, retention of these moorlands was considered to have a high conservation priority.

Heather moorlands supported unusually high populations of Red Grouse. Though many other bird species also utilized this habitat, Red Grouse was the only species entirely restricted to it [26]. However, for most of those who wanted to preserve the moorlands, their retention was motivated not by concern for the ultimate survival of this species but, rather, because Red Grouse shooting was central to local economies. The primary aim of Red Grouse management had always been to maximize the number of individuals available for shooting every Fall. Gamekeepers attempted to achieve this aim through the control of parasites and predators of Red Grouse populations. Among birds, three raptor species were among the implicate predators: the Hen Harrier, the Golden Eagle (*Aquila chrysaetos*), and the Peregrine Falcon (*Falco peregrinus*) [27], [28]. The most important of these (by far) was the Hen Harrier. Hen Harriers, in turn, were prey for Golden Eagles. Though Golden Eagles presumably also preyed on Red Grouse, their role in controlling grouse populations was presumed to be minor compared to that of Hen Harriers [29], [30].

Thirgood *et al.*'s [25] review of the raptor–grouse conflict identified three potential and actual actions that would affect conservation prospects of the three species:

*K*  Hen Harriers could be culled to control their populations. The expected result would be increases in Red Grouse populations and the economic benefits associated with it. Culling was already taking place through hunting which, though technically illegal, was nevertheless apparently widely practiced.
*D*  Diversionary feeding (*e.g.*, carrion) could be introduced for Hen Harriers. This was believed to be able to decrease the predation pressure on Red Grouse though not to the same extent as 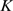. It is assumed in this analysis that this action would benefit Hen Harrier populations to some extent at least so long as culling (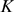) was not undertaken. If culling were introduced, it is likely that 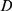 would have very little—if any—effect [31].
*I*  Golden Eagles could be introduced into Hen Harrier habitat. It is assumed (as was very likely) that the benefit to Red Grouse due to Golden Eagle predation of Hen Harriers outweighed the loss due to predation of the Red Grouse. (The analysis below will make the same assumption.)

We next show that each of these potential actions falls under the jurisdiction of a unique agent (an interest group consisting of an easily distinguished set of stakeholders).

#### Agents and Goals

From Thirgood *et al.*'s [25] description, there were many stakeholders involved in the dispute, from gamekeepers whose job was to maintain high Red Grouse populations for hunting, to ardent raptor conservationists interested in either one or both of the raptor species. However, it turns out that these varied stakeholders can be naturally organized into interest groups, each coupled to one of the actions identified above. The principle used for this grouping is that members of each group strongly share interest in some action that the group would encourage and different groups disagree on what that action is. It turns out that, by examining who is likely to be interested in each of the actions discussed above, a natural stratification into three groups becomes possible, simplifying the rest of this analysis.

Thus, each of the following three interest groups will be treated as a single agent in the game-theoretic analysis below:

*A*_1_  Gamekeepers and others who were economically dependent on Red Grouse hunting and wanted their populations to be as large as possible so as to maximize profits from hunting: it is unproblematic to expect that 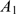 would have control over 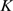 since it is in its interest to cull Hen Harriers. While the other agents may disapprove of culling, it is unlikely that they would have much influence since hunting of Hen Harriers was apparently already being practiced even though it was illegal.
*A*_2_  Hen Harrier conservationists who were concerned primarily with the welfare of that species, in part because they had once disappeared from all of Britain except the Scottish islands of Orkney and Hebrides: 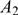 would presumably have almost complete control over 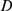, since that action has some potential to help the Hen Harrier population at least when culling does not occur. Moreover, 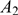 is likely to be the only group willing to expend effort directed towards 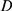 for Hen Harriers.
*A*_3_  Golden Eagle conservationists who were similarly primarily concerned with the welfare of that species. Presumably 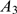 would have sole control over 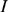 because of its expense, and in spite of probable reservations of 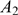, because in carrying out 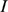, 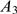 would have at least some support from 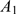.

In one respect this characterization of the interest groups may be slightly artificial since Thirgood *et al.* [25] do not distinguish Hen Harrier and Golden Eagle conservationists quite as sharply. However, it is useful to distinguish them because of the potential for conflict between Hen Harrier and Golden Eagle conservation due to the former being a potential prey of the latter, a problem which Thirgood *et al.* [25] do note.

#### Preference Analysis

Table 2 shows the rank order of the preferences of the agents for each of the eight possible set of three actions that can be taken by the agents. These form the set of alternatives (called “outcomes” in the rest of this paper) in this decision analysis with each action, 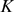, 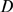, and 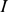 (doing it or not) being an available option for the agent associated with that action. This means that 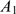 can only choose between 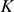 and 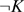, 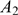 between 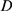 and 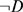, and 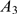 between 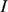 and 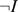. An outcome consists of one action each from each of the three agents (as shown in Table 2), and the complete preference structure consists of a ranking of the entire outcome set by each of the agents.

**Table 2:** Agents' Preference Structure.

	Agent		
Outcome	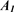	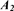	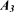
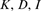	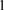	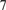	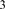
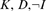	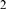	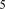	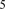
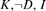	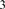	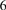	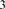
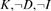	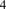	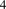	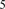
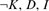	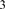	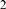	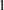
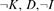	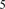	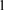	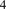
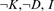	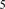	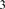	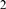
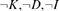	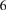	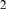	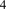

Agents: 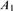: Gamekeepers and Red Grouse hunters; 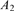: Hen Harrier conservationists; 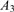: Golden eagle conservationists. Strategies: 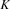: Cull Hen Harriers (or not, 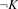); 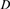: Introduce diversionary feeding for Hen Harriers (or not, 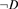); 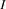: Introduce Golden Eagles into Hen Harrier habitat (or not, 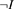). Numbers represent purely ordinal preferences over outcomes (where 1 is most preferred, 2 the next most preferred, and so on). See Appendix S1 for Nash equilibrium analysis and Appendix S2 for Pareto–efficiency analysis. For a justification of the ranking for each stakeholder, see the text.

For 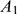, clearly (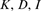) is the best outcome (that is, it has rank 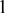), because each of these actions benefit Red Grouse. Assuming that Red Grouse predation by Golden Eagles does happen to some extent (though it is not as serious as culling), the next best outcome is (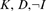). Both (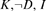) and (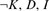) are ranked 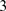, assuming that the combined effect of diversionary feeding and predation and the crucial fact that no effort is expended by 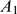 in the latter case cancels out the effect of culling in the former case. Since culling Hen Harriers is potentially a very effective way to reduce Red Grouse mortality (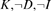) is ranked as 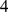. There is probably not much to distinguish (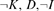) and (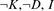)—these are both ranked as 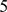. Clearly, (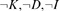) is the worst because no action at all is taken to augment Red Grouse populations.

Agent 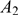
's concerns are limited to Hen Harriers (in this model). Diversionary feeding, along with no culling and no predation, that is, (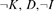), is the best option. Keeping the other two acts as they are, while not introducing diversionary feeding, that is, (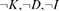) comes in at 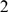 as, from the same type of reasoning, does (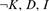). By losing diversionary feeding, (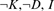) gets rank 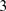. It is assumed that when culling (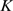) occurs, diversionary feeding (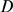) does little to augment Hen Harrier populations, but predation (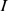) still has a small negative effect on them. Moreover, 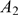 presumably does not want to waste effort in performing 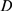 if it does not help Hen Harriers. Thus, taking wasted effort into account, (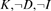) is given rank 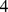, (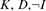) rank 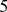, and (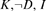) rank 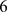. The situation is worst when both culling and predation occur, and 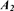 also wastes effort, that is, (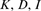).

Turning to 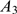, the best outcome for Golden Eagles is clearly (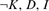), when the species is being introduced in Hen Harrier habitat and the main prey species is being encouraged to grow by no culling and diversionary feeding. For Golden Eagles, the outcome is only slightly worse if Hen Harriers lose diversionary feeding: (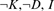) has rank 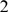. Beyond these two cases, assuming that diversionary feeding is not very important for Hen Harrier populations, the ranks 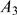 gives will be neutral with respect to 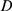 and 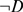. Both (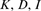) and (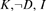) will be ranked 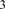. Next come (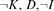) and (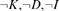). The worst scenarios are (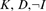) and (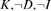).

#### Game–theoretic Analysis

The decision scenario discussed above can be modeled as a three–agent game with each agent, 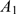, 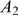, and 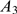 having control over one action: 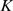, 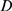, and 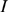, respectively. As noted earlier, this is a simplifying but plausible assumption in this context. Agents' preferences over the eight possible outcomes were enumerated in Table 2. The standard assumptions of one–stage game theory are applicable: each agent has full knowledge of the preference structure and is a competent maximizer over that agent's own preference ordering.

This game will be analyzed to determine which outcomes, if any, are Nash equilibria and which are Pareto–efficient. For simplicity, attention will be restricted to pure strategies. In Appendix S1, it is shown that there is a unique Nash equilibrium, which is the outcome, (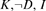). In Appendix S2 it is then shown that there are four Pareto–efficient outcomes, (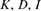), (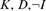), (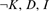), and (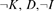). In other words, the Nash equilibrium, (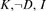), is a Pareto–inefficient outcome. In fact, it is Pareto–inferior to (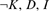), which would leave no agent worse off and 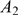 and 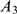 better off.

#### Discussion: Raptors and red grouse

It is worth emphasis that the assumptions about group decisions that are made in computing the set of Pareto–efficient outcomes were minimal. It is only assumed that the outcomes have a complete ranking with ties allowed (that is, a complete weak ordering) on the basis of each agent's preferences. There was no assumption made about whether the outcomes can be given quantitative (cardinal) values. If there is more information available on agents' preferences, more structure can be given to the set of Pareto–efficient outcomes (see below). However, that does not change the existence of the fundamental conflict between Pareto–efficient and Nash equilibrium outcomes because it was generated by the minimal assumptions noted above about what was collectively preferable.

An important limitation of this analysis is that, as in the two–player game discussed earlier, it is restricted to pure strategies: agents do not have the option of mixed strategies in which they sometimes carry out one action and sometimes do not. Moreover, in most practical contexts, a problem remains: there are four Pareto–efficient outcomes: (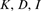), (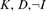), (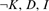), and (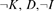) and only one of these can be implemented. The set of Pareto–efficient outcomes may have to be analyzed further to come up with a credible policy recommendation. In the context of multi-criteria decisions in biodiversity conservation planning, this is a well-studied problem [3], [11], [13]. Many of the insights obtained in that context carry over to that of group decisions and the rest of this section draws heavily on those discussions. There are at least two options available at this stage:

1. Additional assumptions about agents' preferences can be introduced to compound them to produce unique results. Methods range from simple voting to aggregating individual utility functions into a group utility function. None of these methods is devoid of conceptual problems which are well–known but beyond the scope of this analysis: they do not resolve the basic conflict between Nash equilibria and Pareto–efficiency.
2. Sorting out the Pareto–efficient alternatives may be handed over to a deliberative process in which the agents discuss these outcomes. (If the number of Pareto–efficient outcomes is small—say, less than five—this is a far more appropriate response than if it is large. In general the number of these outcomes will scale with the number of agents [32]. Some criteria that may be used (but are not immune to the charge of being *ad hoc*) have some reasonable intuitive support. For instance, any extremal outcome (a unique outcome that is the most preferred by any of the agents) will always be Pareto–efficient no matter how poorly it is ranked by all other agents. It may, therefore, be reasonable to drop most of such extremal outcomes by deliberative choice: in the case study of this paper, (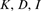), (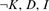), and (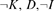) would be dropped leaving only (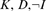) as a policy recommendation. Another method may be to deliberate on the values of all agents. In the case study here, it is reasonable to suppose that 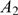 and 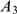 may have moral scruples about killing animals. Thus, they may want to drop (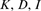) and (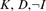) and then agree to choose (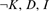) over (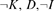) because that is in accord with 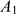
's preferences.

The most important conclusion suggested by our discussion is that the best way to resolve the conflict between the Nash equilibrium and Pareto–efficiency is through a deliberative process and not through mechanism–design, that is, the elaboration of individual material incentives. While it is plausible to alter incentives to change the preference structure of Table 2 to remove this conflict, the fact that it occurred from a straightforward preference set attribution suggests that such conflicts will be ubiquitous. This is why we emphasize deliberative collective decisions rather than mechanism–design. Finally, it should also be noted that, unlike the two–agent wild dogs case presented above, this game is not straightforwardly interpretable as a Prisoner's Dilemma event though it shares with that game the property of having a unique Nash equilibrium that is also Pareto–inefficient.

### The *n*–agent dilemma: Fish and corals in the Philippines

Coral reefs, especially those in the southern Philippines and central Indonesia, are widely regarded as biodiversity “hotspots” of high conservation priority [33]. These rich marine ecosystems are home to hundreds of thousands of fish, bivalve, gastropod, cephalopod, crustacean, echinoderm, algae, and other species, many of which are typically micro–endemics. While human activities on land contribute to reef degradation via the “downstream” effects of agricultural and logging activities, industrial run-offs and other pollutants, in the marine arena, overfishing and destructive fishing techniques (*e.g.*, those using improvised explosives or sodium cyanide) have also been centrally implicated in reef destruction [33]. These reefs are often vital to local economies. In the Philippines, for example, over–crowded coral fisheries support an economic livelihood for over a million fishers [34].

The destructive ecological effects of overfishing on coral reefs are well documented. Two examples will help set the context [35]: (i) in the Philippine coral reef system of Bolinao, overfishing led to near extinction for the sea urchin (*Tripneustis gratilla*), which had been formerly quite abundant in the reef's seagrass beds; (ii) in Kenya reefs were threatened by overfishing because the removal of high–level predators led to a dramatic increase in populations of drupellid snails which feed on coral.

According to McManus *et al.* [36], roughly 350 marine species from the 40 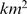 Bolinao reef area are sold in local markets. In spite of the practice being banned in 1979, fishers continue to use explosive fishing techniques and have a strong financial incentive to do so: dangerous homemade bombs are cheap to produce at US $1–2 and can generate a catch worth US $15–40 while the average fisher, using non–destructive techniques, generates only about US $1 a day. They report that informal surveys of the reef area in the mid–1980s showed that 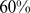 of scleractinian coral was dead, much apparently due to fishing with explosives. Furthermore, their simple models indicated that fishing with explosives may have reduced the growth capacity of scleractinian coral by a third or more, with predictably negative effects for biodiversity.

Game–theoretic analyses have been used in many analyses of fishing policies (and these models have been reviewed by Sumaila [4]). The open–access version of the 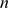
–agent game described below corrsponds to the classic “tragedy of the commons.” While the analysis is simple, we provide it because it captures the dynamics of overfishing in coral reefs in Bolinao where the resource is over–exploited because of no clear established rights of use. The Nash equilibrium outcome of collective over–exploitation of fish and the use of destructive fishing techniques is both economically undesirable (because of Pareto–inefficiency), as well as a major threat to healthy reefs and, thus, to sustainability and the conservation of biodiversity. However, we then go on to show that, even in a closed–access 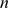
–agent gave, there can be a conflict between resource management policies based on “maximum sustainable yield” (MSY) and biodiversity conservation. In this case, due to the ecological interactions between the exploited fish and other reef species of high conservation priority, MSY harvest levels for the exploited species may lead to a decline of other species targeted for conservation as important components of biodiversity. The analysis assumes that, in the long run, this trend leads to a decline in the exploited species because of mutualistic interactions–however, this part of the analysis should be regarded as a conceptual exercise rather than an exploration of the data.

#### Game-theoretic Analysis

The Gordon–Schaefer model of open–access fisheries [37], [38], as well as Hardin's [39] less formal “tragedy of the commons” model of common pool resources, predict overfishing when individual or collective access/property rights to fisheries are ill–defined. The “bionomic equilibrium” is the point at which the population is so depleted that even minimal harvesting effort is not worth the expected return [40].

This situation can also be represented as an *n*–agent PD, with the payoffs for agents along the rows as given in Table 3. The payoffs are symmetric for all agents, in the sense that *all agents* find themselves in the situation described by the payoff matrix. The non–cooperative action, 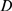, is to harvest as much as possible now (or, in the case of overfishing in Philippine reefs, use destructive fishing methods like dynamite or cyanide). 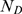 denotes the number of agents who play 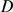, the non-cooperative harvesting effort, and 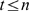 is some threshold value (“tipping point”) such that, where 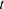 or more agents defect, the outcome shifts from the left to the right column: the common–pool resource is overexploited and fishing is not worth the effort. We assume the cooperative action 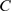 is to restrain harvesting effort to a level such that, if 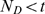, the population is sustainable over time.

**Table 3:** Open-access *n*–agent game.

	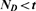	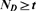
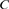	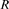	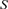
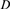	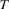	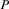

Agents: 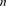 fishers in an open–access fishery. Strategies: 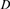: harvest as much as possible now; 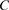: restrain harvesting effort to maximum sustainable yield levels. 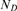: number of agents who play 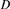, 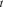 is tipping point where harvesting effort exceeds maximum sustainable yield levels. It is assumed that 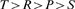 for each fisher.

For the preference structure, we assume 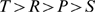. This means that the the worst case for each agents is to restrain harvesting effort while others overexploit the fish. The best case is for an insignificant number of others (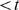) to defect while agents harvests as much as possible. The second–best case for agents is to cooperate while all (or a significant number) of others cooperate by restraining harvesting effort. The third–best case for an agent is to defect, achieving a short–term gain while the fish population reaches bionomic equilibrium: enough agents defect such that the population is over–exploited in a short time. An agent does better by defecting no matter what the others do, since 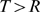 and 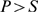. The Nash equilibrium solution of this game is the situation in which all defect, and the fish are overexploited. This is each agent's third–best outcome, whereas if everyone cooperated they would have achieved their second–best outcomes, and the exploited population of fish would persist at a sustainable level.

In this case, biodiversity values and economic values both prescribe conservation action. Economically, the open–access Nash equilibrium is inferior to the cooperative outcome for every agent: the latter is strictly preferred to the former by every agent. Furthermore, the destructive fishing techniques and overfishing that characterize the open–access equilibrium clearly threaten reef integrity and biodiversity.

However, a second *n*–agent PD may arise that pits economic and conservation values against one another in the short term. Consider the situation in which the open–access problem (the “tragedy of the commons”) for some reef fishery has been solved by privatization, government control, or community management, such that a resource management plan for “maximum sustainable yield” (MSY) of the fish has been instituted. We still assume there are a number of agents extracting fish, but in this game the Nash equilibrium is for the agents to “restrain” their harvesting effort to the MSY level. Crucially, we make an assumption about the ecological interactions between the exploited fish and the surrounding reef ecosystem: the MSY harvesting effort will, over time, lead to a slow decline in some endemic species of high conservation priority. As the population of this second species declines, the population of the exploited species will as well, such that the “MSY” harvesting effort is actually unsustainable. (For a partial justification of this assumption, see the cases described by Redford and Feinsinger [41].)

This second, parallel *n*–agent PD is represented in Table 4. In the open–access 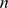
–agent game, we assumed that some policy similar to MSY harvesting was the “cooperative” option. In the closed–access case, the cooperative action will be denoted by 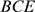, for biodiversity conservation effort, and the non–cooperative option is the more intensive MSY harvesting, denoted by 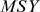. Otherwise, the preference structure is exactly parallel. The Nash equilibrium solution sustains the fish species in the short term, but as the second species slowly declines, in the long run, catches of the economically valuable fish decline in turn. Thus the Pareto–efficient solution involves each agent restraining harvesting effort beyond the short–term MSY point.

**Table 4:** Closed–access *n*–agent game.

	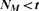	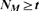
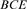	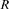	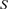
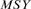	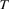	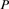

Agents: 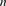 fishers in a closed–access fishery. Strategies: 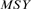: harvest at maximum sustainable yield levels; 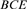: restrain harvesting effort to long-term biodiversity-promoting levels. 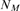: number of agents who play 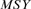, 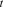 is tipping point where harvesting effort leads to eventual decline in yield due to ecological interaction with species of conservation value. It is assumed that 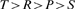 for each fisher.

#### Discussion: Fish and corals in Southeast Asia

Overfishing in coral reefs is both an economic and biodiversity conservation issue, especially in cases like that described by White *et al.* [34], in which fish levels in some areas of the Philippines have dropped below those necessary to sustain healthy coral reefs. They report that while healthy reefs can sustainably produce 20 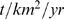 of edible products, reefs degraded due to overfishing or cyanide use produce less than 4 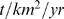. (Other economic benefits attaching to the preservation of biodiversity in reefs include revenue from tourism: reef diving, tour fees, *etc.*)

Admittedly, our closed–access 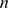
–agent PD is only a speculative ecological model, but it brings into focus the need for conservation and resource management planners to take long–term ecological interactions into account in assessing solutions to conservation–relevant economic conflicts. In particuar, it shows that appeal to theories of sustainable exploitation may at best produce short–term Pareto optimal outcomes.

The formal mechanism–design solution to the 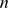
–agent PD alters the preference structure for each agent *via* material incentives or the threat of punishment by defining clear use rights. It is well–known that enforceable government ownership, group ownership, or individual ownership can go a long way towards preventing over–exploitation of resources [42]. Even in closed–access fisheries, however, further regulations and incentives may be necessary to ensure sustainability when multiple users compete [43]. While such formal solutions can be effective, two comments are in order. First, resource users can and do develop informal networks of trust and reciprocity norms that can solve open access dilemmas [44]. Second, appealing solely to agents' self–regarding preferences can be counterproductive. In the discussion below we consider evidence that suggests limitations of such narrow mechanism–design solutions.

## Discussion

What should happen when Nash equilibria are Pareto–inefficient? The answer is simple: agents should cooperate. However, willingness to do so depends on the level of trust, more specifically, the degree of confidence an agent has that another agent will not unilaterally change strategy. But this is what is required to make Nash equilibria of games irrelevant in the sense that agents would not have to worry about the possibility that some agent will act without consideration of the others. How can such trust be built? The obvious suggestion is more discussion and deliberation and, especially, confidence–building measures in these situations. In environmental decision contexts, given that few agents actively claim an explicit desire to harm environmental goods and services, collective decision–making through deliberation is the obvious recommendation. In the South African case study, the pursuit of such deliberative strategies should presumably include conservation education and credible plans from conservation proponents to offset costs incurred by herders and game farmers due to predation by wild dogs. This recommendation is easy to make because only two agents are involved. The situation in the British example is more complex, requiring reciprocal commitments between gamekeepers and the two classes of raptor conservationists. For instance, if each of the three agents agreed to drop a policy which is deemed best by only one agent, there would remain only one outcome, (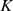, 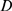, 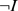), on which they would have to agree. In the case of overfishing on coral reefs in the Philippines, collective deliberation would presumably have to take place through public forums because of the number of agents that are involved.

The contrast is with the traditional mechanism–design strategy for achieving Pareto–efficient outcomes in games like those presented earlier. The preferred strategy has been to alter *material incentives*, on the assumption that self–interested actors will respond to those incentives. These actors are assumed to be (or to closely resemble) the (in)famous *Homo economicus* [45], [46] of rational–choice economic models, including traditional game theory, supplemented by substantive assumptions about preferences over material goods. Bowles [6] has noted this mechanism–design view makes strong and controversial foundational assumptions. Narrow self–interest is presumed to be the basis for social institutions and resulting institutional arrangements. Insitutional policies are supposed to work best when designed for “knaves.”

While policies based on mechanism–design incentives have some record of success, recent experimental results in behavioral economics show that there are significant limitations to narrow mechanism–design solutions [6], [46] that are particularly relevant in planning for environmental values, including biodiversity conservation. In certain types of situation this style of solution might actually backfire, by *undermining* the “moral sentiments” that can contribute to cooperative behavior. In short, appeal to narrow (material) self–interest may “crowd out” other–regarding motives. Briefly, these types of limitations include the following [6]:

1. Framing and informational effects: Where cooperation is “framed” (in the psychologist's sense) as required by regulation or law, and enforced, *e.g.*, by a fine, this may actually undermine cooperative behavior over time. It may be better to frame cooperation in the context of group decision–making amidst informal networks of communication, appealing to agents' other-regarding motives [47]. Further, material incentives may send a negative signal to the agents that can motivate defection, for instance, because they may indicate a lack of trust [48].
2. Learning effects: Incentives may provide an environment in which agents “learn” to be more self–interested, and their preferences shift over time to become less other–regarding [49].
3. Overdetermination: A significant body of psychological research on “intrinsic” motivations suggests that, when agents are offered financial incentives for actions for which they are already intrinsically motivated (*e.g.*, because they are pleasurable), intrinsic motivation may decrease significantly [50]–[54].

When these kinds of situations obtain, deliberative (rather than formal) solutions that engage the “moral sentiments” (other-regarding motives) of the agents are likely to better achieve the goals envisioned by them. Encouraging agents to communicate and reach agreements on behavior, for instance, through credible promises of future behavior to each other, may be sufficient. If agents are sure that others will not unilaterally change their actions, the Nash equilibrium becomes irrelevant. Even in the absence of explicit agreements, if each agent were aware of the value structures of the other agents, that might provide reasonable ground for expecting that other agents will not change behavior in certain ways. Additionally, an agent may use knowledge of other agents' values to adjust the agent's own courses of action. In both of these cases, Nash equilibria may become irrelevant.

An important limitation of these analyses is their restriction to pure strategies: agents do not have the option of mixed strategies in which they sometimes carry out one action and sometimes do not. Whether mixed strategies would also lead to the problems noted here—and to what extent—remains an open question to be explored on some other occasion. However, in the practical context of conservation decisions, it is unlikely that any agent is likely to follow a mixed strategy.

A final methodological point will conclude this paper. Though game theory has been used in other environmental decision contexts [4], it has very rarely been used in the context of decision analysis for biodiversity conservation (and, even then, only in the context of two–agent games. [5], [55]). (With respect to biodiversity policy, it has more often been used in contexts such as that of stakeholder disagreement about intellectual property rights [56].) The analysis here shows that it is relatively easy to use game theory to identify situations in which the pursuit of individual self–interest using whatever incentive set that is in place leads to sub-optimal outcomes from the perspective of minimal group interests. Typically, unlike almost all uses of game theory in environmental decision contexts, the analysis will require multi-agent (rather than two–agent) models. However, as this analysis shows, these models may still not be intractably complex and can be fairly easily analyzed. The most important point is that game theory can be used as a normative tool to identify situations in which members of groups (agents) should be encouraged to interact, communicate, and deliberate jointly because the initial preference structure is such that the Nash equilibria are definitively sub-optimal from the point of view of what is desirable for a group.

## Methods

This section briefly introduces the methodological framework of decision analysis and game theory. The first subsection introduces the case where a single agent makes a decision over a set of feasible alternatives. The second introduces interdependent decision problems (games), two standard criteria by which solutions are evaluated, Nash equilibria and Pareto–efficiency, and an example of a game with a Pareto–inefficient Nash equilibrium (the Prisoner's Dilemma).

### Rational choice: a single agent

In the simplest case of rational decisions under certainty, a single agent chooses between a set of feasible alternatives 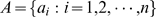, which are all (weakly) ranked according to a single evaluative criterion 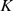. The ranking 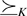 is a weak order since indifference is allowed: 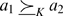 means that 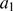 is at least as preferred as 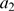 and if, additionally, 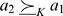 holds, then the agent is indifferent between 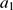 and 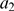. Strict preference 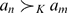 can be defined as 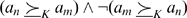. Additionally, we require the ranking 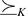 to satisfy the following constraints:

1. *Completeness*: all alternatives are ranked. 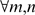 either 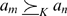 or 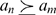.
2. *Symmetry*: all alternatives are at least as preferred as themselves: 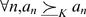.
3. *Transitivity*: if some alternative is weakly preferred to a second, and the second to a third, the first is weakly preferred to the third: 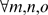, if 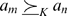 and 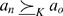 then 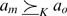.

In the context of biodiversity consrvation planning, for instance, the set of alternatives might be potential conservation areas and the evaluative criterion some operational measure of biodiversity value [57]. We assume our goal is to choose the most preferred alternative among the feasible ones. In such a simple case, the rational choice is clearly an alternative 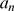 such that 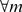, 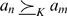, that is, 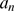 is at least weakly preferred to all other alternatives. Or, equivalently, we choose the alternative 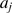 such that 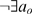 such that 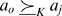 and 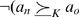), that is, there does not exist an alternative that is strictly preferred to 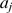. There may be more than one alternative that satisfy these conditions, in which case we have a tie for first place but, in this simple example, no information to break such ties.

All of the agents in our game–theoretic examples exemplify this simple case of decision over a single criterion–based ordinal ranking under certainty, but with the added complication that outcomes depend on the choices of other agents (see below). However, more complicated decision scenarios may involve:

**Multiple criteria:** For many decision problems, there may be multiple criteria of evaluation, producing rankings over alternatives that may be in conflict. Various methods have been devised for rational decision-making with multiple criteria [58]. For a discussion of multi–criteria analysis in the context of biodiversity conservation, see Moffett and Sarkar [3].
**Cardinal utility:** If information is available about *how much* some alternatives are preferred to others, *e.g.*, by using the von Neumann and Morgenstern [1] method of eliciting preferences over gambles, then a real-valued “utility” function 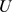 may be constructed that maps alternatives to cardinal values. Utility is then a numerical measure of preference.
**Uncertainty:** Many (perhaps most) decisions are made in the context of uncertainty. Suppose that the probability of the consequences of a decision are (causally and epistemically) independent of which alternative is chosen, and that probabilities can be assigned to those consequences. Then rational choice maximizes “expected utility,” where the expected utility of an action is a probability–weighted sum of its utility values in the various outcomes.

### Interdependent decisions: game theory

#### Definitions

Game theory can be used to model situations of interdependent decisions, where multiple agents choose from their own feasible set of alternatives (actions), and the outcome depends on the choices of all the agents. Agents' preferences are over the outcomes, which are complete specifications of each of the agents' actions. Thus we have a set of agents 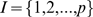, a set of feasible actions for each agent 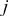, 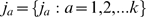, and a set 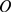 of possible outcomes 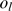, which are the possible 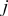
–tuples of actions 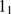 through 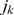. We assume for each agent 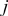, a ranking 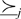 over the outcomes in 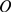. These rankings satisfy the constraints listed earlier. Thus we make the simplifying assumption that each agent has a single criterion ranking over the outcomes. Games with multiple agents and multiple criteria are possible but not explored here.

An outcome is *Pareto*–*efficient* if and only if no agent could be made better off without making another agent worse off. Thus some outcome 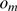 is Pareto–efficient if and only if 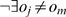 such that 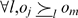 *and* 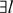 such that 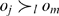. Pareto–efficiency is a weak criterion, since any outcome most preferred by some agent is *ipso facto* Pareto–efficient.

The Nash equilibrium [59], is that outcome such that no agent can do better by *unilaterally* deviating from an existing pattern of action. In other words, holding the strategies of the others fixed, if no agent can change strategy and do better, the outcome is in equilibrium. Thus some outcome 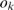 is a Nash equilibrium if and only if there does not exist a 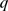 such that there exists an alternative action 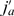, keeping all other agents' actions in 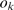 fixed, leading to outcome 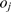 such that 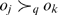. The sense in which this is an “equilibrium” is that no agent has an incentive to change strategy *unilaterally*.

Finally, there is a distinction between one–off (static) and iterated (dynamic) games. A game like the simple one above may be played once, or multiple times, either between the same individuals or between different individuals. The iterated version allows for more interesting kinds of strategies, *e.g.*, conditional strategies, that is, an agent may choose an action in response to other agents' actions in the previous step. Notably, iterated games provide the basis for *evolutionary game theory*. Evolutionary game theory gives up economists' strong assumptions about agent rationality. Agents in a population use fixed strategies and the success of those strategies determines their frequency in the next time step. Evolutionary game theory models frequency–dependent selection, where the fitness (payoff) of some strategy depends on the composition of the population.

The representational power and flexibility of game theory will be seen in the following two examples. The first example will illustrate game theory in perhaps the simplest of cases. The second is the best–known example of games with Pareto–inefficient Nash equilibria. We include it because we refer to it several times, for instance, in connection to the case of wild dog conservation in South Africa.

#### Driving coordination game

Consider first the two–agent “coordination” game of “driving” in Table 5, where each agent either drives on the right or the left. Each agent strictly prefers the outcomes where both drive on the right or the left to the (disastrous) outcomes where their actions differ, but is indifferent between the first pair of outcomes and the latter pair. The game can be represented in “normal form” as a table like this one, where the preferences are given 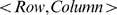 and 1 is the rank of the most preferred outcomes and 2 that of less preferred ones.

**Table 5:** Driving coordination game.

	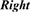	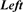
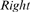	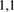	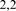
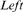	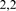	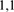

Row and Column decide whether to drive on the 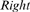 or 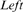. Numbers represent purely ordinal preferences over outcomes (where 1 is most preferred, and so on), and are given 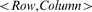.

In the driving example, both the 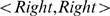 and the 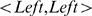 outcomes satisfy the criterion of Pareto–efficiency, since no agent can be made better off without making at least one agent worse off—in fact, both would be worse off in the 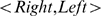 or 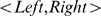 outcomes. Furthermore, both the 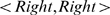 and the 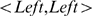 outcomes are Nash equilibria. No agent has an incentive to unilaterally deviate from playing 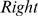 (*Left*), for example, if the other agent is also playing 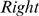 (*Left*).

#### Prisoner's Dilemma

The game in Table 6 is a generalized ordinal formulation of the Prisoner's Dilemma as a two–agent game, where 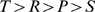 for each agent. As a useful mnemonic, these letters are usually taken to stand for temptation, reward, punishment, and sucker, respectively. An agent corresponding to a row and one corresponding between the row choose between cooperation (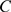) and defection (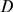). Payoffs are given 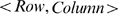 in the matrix. In the standard quantitative formulation of PD, where the ranks are interpreted as numbers, there is an additional requirement: 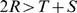.

**Table 6:** The Prisoner's Dilemma.

	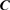	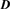
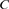	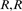	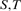
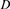	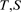	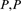

Row and Column decide whether to cooperate (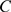) or defect (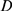). The preference structure is ordinally given by 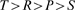, where, if the numbers can be interpreted quantitatively, 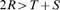.

The original story of the prisoner's dilemma involves two prisoners, separated by the police, whose options are to confess (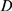) or to stay silent (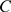). If both stay silent, then both get a light prison sentence (reward); if both confess, then both get a moderate prison sentence (punishment). However if one confesses and one stays silent, the confessor gets off without a prison sentence (temptation), while the one who stayed silent gets a heavy prison sentence (sucker). Each prisoner does better by confessing, no matter what the other does. But each prisoner confessing leads to a worse outcome for both prisoners than if they had both stayed silent.

The game is of significant interest because its Nash equilibrium, 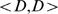, is Pareto–inefficient a scan be seen by comparing to the mutually cooperative outcome, 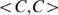. It is easy to see that each agent does better defecting, no matter what the other agent does, since 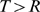 and 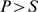. However, each agent could be made better off by switching to the cooperative outcome, since 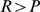. However, the cooperative outcome 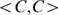 is unstable, since either agent could do better individually by switching to 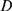, since 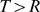.

All the game–theoretic models analyzed in this paper share the property that the Nash equilibria are Pareto–inefficient.

## Supporting Information

Appendix S1 Nash equilibrium for three-agent raptors and red grouse dilemma.
(0.05 MB PDF)

Appendix S2 Pareto-efficient outcomes for three-agent raptors and red grouse dilemma.
(0.05 MB PDF)
