# Supplementary material for: Group Decisions in Biodiversity Conservation: Implications from Game Theory
Source: PLoS One. 2010 May 27;5(5):e10688. doi: 10.1371/journal.pone.0010688 (PMC2877714; doi:10.1371/journal.pone.0010688)
Supplement: Appendix S1 — Nash equilibrium for three-agent raptors and red grouse dilemma. (0.05 MB PDF) [file pone.0010688.s001.pdf]

Group Decisions in Biodiversity Conservation: Implications from Game Theory

David M. Frank[1], Sahotra Sarkar[2,*]
1 Department of Philosophy, University of Texas, Austin TX, USA
2 Section of Integrative Biology and Department of Philosophy, University of Texas, Austin TX, USA
$*$ **E-mail: sarkar@mail.utexas.edu**

Appendix S1: Nash equilibrium for three-agent raptors and red grouse dilemma

Since there are only eight outcomes, it is straightforward to determine whether each of them is a Nash equilibrium sequentially, as is done in the table below. Each outcome is tested for its stability, that is whether any deviation from it on the part of one of the agents would result in a more preferred outcome for that agent. Outcomes are labeled as "unstable" and the others as "stable," with the stable outcomes being the Nash equilibria. By inspection, it is found that $(K, \neg D, I)$ is the unique Nash equilibrium for this game.

Table 1. The Nash Equilibrium. Agents: A_1: Gamekeepers and Red Grouse hunters; A_2: Hen Harrier conservationists; A_3: Golden eagle conservationists. Strategies: K: Cull Hen Harriers (or not, $\neg K$); D: Introduce diversionary feeding for Hen Harriers (or not, $\neg D$); I: Introduce Golden Eagles into Hen Harrier habitat (or not, $\neg I$). This table enumerates all the outcomes and shows why only $(K, \neg D, I)$ is a Nash equilibrium.

**Outcome**	**Stability**	**Analysis**
$K,\ D,\ I$	Unstable	A_2 can unilaterally deviate to $(K, \neg D, I)$, ranked 6 instead of 7.
$K,\ D, \neg I$	Unstable	A_2 can unilaterally deviate to $(K, \neg D, \neg I)$, ranked 4 instead of 5. A_3 also has an incentive to deviate unilaterally to (K, D, I), ranked 3 instead of 5.
$K, \neg D,\ I$	Stable	No agent has an incentive to deviate unilaterally. This is the Nash equilibrium. Consider each ag -ent's possible unilateral deviations. A deviation by A_1 would result in $(\neg K, \neg D, I)$, ranked worse, 5, instead of 3. A deviation by A_2 would result in (K, D, I), ranked worse, 7, instead of 6. A deviation by A_3 would result in $(K, \neg D, \neg I)$, ranked worse, 5, instead of 3.
$K, \neg D, \neg I$	Unstable	A_3 can unilaterally deviate to $(K, \neg D, I)$, ranked 3 instead of 5.
$\neg K,\ D,\ I$	Unstable	A_1 can unilaterally deviate to (K, D, I), ranked 1 instead of 3.
$\neg K,\ D, \neg I$	Unstable	A_1 can unilaterally deviate to $(K, D, \neg I)$, ranked 2 instead of 5. A_3 can also unilaterally deviate to $(\neg K, D, I)$, ranked 1 instead of 4.
$\neg K, \neg D,\ I$	Unstable	A_1 can unilaterally deviate to $(K, \neg D, I)$, ranked 3 instead of 5. A_2 can also unilaterally deviate to to $(\neg K, D, I)$, ranked 2 instead of 3.
$\neg K, \neg D, \neg I$	Unstable	A_1 can unilaterally deviate to $(K, \neg D, \neg I)$, ranked 4 instead of 6. A_2 can also unilaterally deviate to $(\neg K, D, \neg I)$, ranked 1 instead of 2. A_3 can also unilaterally deviate to $(\neg K, \neg D, I)$, ranked 2 instead of 4.
