# Supplementary material for: Group Decisions in Biodiversity Conservation: Implications from Game Theory
Source: PLoS One. 2010 May 27;5(5):e10688. doi: 10.1371/journal.pone.0010688 (PMC2877714; doi:10.1371/journal.pone.0010688)
Supplement: Appendix S2 — Pareto-efficient outcomes for three-agent raptors and red grouse dilemma. (0.05 MB PDF) [file pone.0010688.s002.pdf]

Group Decisions in Biodiversity Conservation: Implications from Game Theory

David M. Frank[1], Sahotra Sarkar[2,*]
1 Department of Philosophy, University of Texas, Austin TX, USA
2 Section of Integrative Biology and Department of Philosophy, University of Texas, Austin TX, USA
$*$ **E-mail: sarkar@mail.utexas.edu**

Appendix S2: Pareto–efficient outcomes for three-agent raptors and red grouse dilemma

Once again, since there are only eight outcomes, each can be analyzed for Pareto–efficiency. However, note that every "extremal" oucome, that is, a unique outcome that is the most preferred by any of the agents is bound to be Pareto–efficient because every other outcome is less preferred by that agent. In this example, this rule can be used immediately to identify (K, D, I), $(\neg K, D, I)$, and $(\neg K, D, \neg I)$ as Pareto–efficient. The remaining five outcomes were then investigated in more detail to produce the results in the table below.

Table 1. Pareto–efficient Outcomes for Three–agent Raptors and Red Grouse Dilemma. Agents: A_1: Gamekeepers and Red Grouse hunters; A_2: Hen Harrier conservationists; A_3: Golden eagle conservationists. Strategies: K: Cull Hen Harriers (or not, $\neg K$); D: Introduce diversionary feeding for Hen Harriers (or not, $\neg D$); I: Introduce Golden Eagles into Hen Harrier habitat (or not, $\neg I$).This table enumerates all the outcomes and analyzes each with respect to Pareto–efficiency. I: Pareto–inefficient; E: Pareto–efficient.

**Outcome**	**Efficiency**	**Analysis**
$K,\ D,\ I$	E	Since this is A_1's unique best outcome, switching outcomes could only make other agents better off by making A_1 worse off.
$K,\ D, \neg I$	E	A_1 and A_3 would be better off by switching to (K, D, I), but this would make A_2 worse off. Any other switch would make A_1 worse off.
$K, \neg D,\ I$	I	A switch to $(\neg K, D, I)$, which would make A_2 and A_3 better off, while leaving A_1 with the same rank of 3. Note that this is the unique Nash equilibrium.
$K, \neg D, \neg I$	I	All agents could be made better off by switching to $(\neg K, D, I)$: A_1 from rank 4 to 3, A_2 from rank 4 to 2, and A_3 from rank 5 to 1.
$\neg K,\ D,\ I$	E	It is A_3's unique best outcome, so by the same reasoning as above, it is trivially Pareto–efficient.
$\neg K,\ D, \neg I$	E	This is A_2's unique best outcome.
$\neg K, \neg D,\ I$	I	A switch to $(\neg K, D, I)$ would make everyone better off: A_1 from rank 5 to 3, A_2 from rank 3 to 2, and A_3 from rank 2 to 1.
$\neg K, \neg D, \neg I$	I	A switch to $(\neg K, D, I)$ would make everyone at least as well off: A_1 from rank 6 to 3, A_2 stays at rank 2, and A_3 from rank 4 to 1.
